# Performing collaborative research: a dramaturgical reflection on an institutional knowledge brokering service in the North East of England

**DOI:** 10.1186/s12961-019-0449-7

**Published:** 2019-05-08

**Authors:** Peter van der Graaf, Janet Shucksmith, Rosemary Rushmer, Avril Rhodes, Mark Welford

**Affiliations:** Teesside University, School of Health and Social Care, Constantine Building, Middlesbrough, TS1 3BA United Kingdom

**Keywords:** Institutional knowledge brokering, public health, Goffman, auto-ethnography

## Abstract

**Background:**

To increase the uptake of research evidence in practice, responsive research services have been developed within universities that broker access to academic expertise for practitioners and decision-makers. However, there has been little examination of the process of knowledge brokering within these services. This paper reflects on this process within the AskFuse service, which was launched in June 2013 by Fuse, the Centre for Translational Research in Public Health, in North East England. The paper outlines the challenges and opportunities faced by both academics and health practitioners collaborating through the service.

**Methods:**

The authors reflected on conversations between the AskFuse Research Manager and policy and practice partners accessing the service between June 2013 and March 2017. Summary notes of these conversations, including emails and documents relating to over 240 enquiries, have been analysed using an auto-ethnographic approach.

**Findings:**

We identified five challenges to knowledge brokering in an institutional service, namely length of brokerage time required, limits to collaboration, lack of resources, brokering research in a changing system, and multiple types of knowledge.

**Conclusions:**

To understand and overcome some of the identified challenges, we employ Goffman’s dramaturgical perspective and argue for making better use of the distinction between front and back stages in the knowledge brokering process. We emphasise the importance of back stages for defusing destructive information that could discredit collaborative performances.

## Background

### Knowledge brokering: opportunities and challenges

The need for closer interaction between those working in public health policy and practice and researchers has long been recognised [1]. However, the ways that public health practitioners can effectively relate to, interact with, undertake and commission research from university researchers to support the development of evidence-based practice are not clear. The difficulties for collaborative research have been well documented in previous research [2–4] and suggest a need for opportunities and spaces for researchers and public health practitioners to work together to generate research findings of greater utility to public health practice. Employing individual knowledge brokers may not be sufficient to mobilise research evidence in the day-to-day practice [5]; instead, a collective process of ‘brokering’ at the organisational level may be required. Studies of university-based knowledge brokering have highlighted issues of sustainability in how knowledge broker roles are resourced and structured within universities, with many knowledge brokers faced with challenges such as short-term contracts, limited career progression and competing demands alongside their knowledge broker role [6, 7].

Several universities have experimented with new services and programmes to create these opportunities and spaces. For example, York University in Toronto, Canada, developed a dedicated Knowledge Mobilization Unit to promote better linkages between research producers and users, particularly in local communities [8]. Additionally, the Centre for Research on Families and Relationships was established between several Scottish universities at least partly to improve research use and linkages between research and policy [9].

Fuse was established in 2008 as one of five public health research centres of excellence in the United Kingdom, funded by the United Kingdom Clinical Research Collaboration. A prime focus of the Centre is the translation of the research produced into usable evidence. In June 2013, after extensive consultation with local stakeholders and partners [10], Fuse launched AskFuse to provide policy-makers and practitioners with an easy-to-access portal for public health evidence and as a rapid response and evaluation service for policy and practice partners in the North East of England.

Herein, we will describe the knowledge brokering process through AskFuse and highlight the challenges and opportunities faced by both academics and the policy and practice partners collaborating through the service, summarised in five themes. These themes are in line with findings of other research on knowledge broker services. What is new in this paper is the theoretical lens [11] that we apply to make sense of these challenges and the solutions suggested by this lens for addressing these challenges.

### Applying a dramaturgical lens

Our starting position is not to try and reduce the gaps between policy, practice and research but to use these gaps more strategically, as suggested by Wehrens [12] in his study of the Dutch Academic Collaborative Centres for Public Health. He proposed a distinction between a ‘front and back stage’ in partnerships and studying the performances on these separate stages at different times in the collaboration process.

These concepts borrow from Erving Goffman’s dramaturgical perspective on social interaction. Goffman [13] describes a front stage as a space where a performance is given; in this case academics presenting their research findings, health practitioners developing and delivering interventions, and policy-makers prioritising spending and commissioning interventions. The aim of each performance is to dramatize a reality for an audience; for instance, academics need to emphasise the rigour, objectivity and independent status of their research, while practitioners will emphasise how an intervention will improve health delivery and quality of care for patients. Decision-makers are more likely to make a show of the value-for-money that an intervention will provide and how it will benefit local people. In contrast, Goffman defines a back stage as an area that is off-limits to the audience and therefore provides a safe haven for the performers to relax, drop their public persona and step out of character [11]. In the Dutch example, the content of the academic reports and the research process were intensely debated behind the scenes in various back stage settings between academics, health professionals and policy-makers to ensure that the research objectives and findings were embedded in the wider political context. In other words, collaboration and distinction were highlighted at different points in the co-production process to enable each community to explain and sell their work to their peers.

The distinction between front and back stages may thus provide a useful perspective for analysing the process of knowledge brokering between academia, policy and practice. Goffman’s dramaturgical perspective allows for conceptualising knowledge brokering simultaneously as a process of boundary maintenance in front of stage areas and boundary blurring in the back stage areas of collaborative research. The need for boundary blurring has been emphasised as an important mechanism for knowledge exchange [13]. What Wehrens [12], and by extension Goffman, add to this approach is a simultaneous process of boundary maintenance between academia and policy-makers as a mechanism to establish credibility in both worlds.

### Aim/contribution of this paper

Herein, we apply Goffman’s front and back stage analogy to the knowledge brokering process embedded in our rapid response research enquiry service called AskFuse. By applying Goffman’s dramaturgical perspective to our experiences of the service, we illustrate the functions and value of front and back stages in knowledge brokering.

## Methods

Data is drawn from conversations with policy and practice partners as part of the scoping of enquiries that the service received between June 2013 and March 2017. Individual conversations with over 150 enquirers and academic supporters were documented in summary notes (mostly handwritten on paper) and all email communications with both were stored in separate data folders on the University servers to keep a record of these conversations. More than a quarter of enquiries (27%) originated from public health teams in local authorities who wanted to rapidly evaluate their interventions and programmes; another quarter (23%) was submitted by voluntary and community sector organisations that provided public health-related services in their communities (e.g. social activities for elderly people living in isolation), while a fifth (21%) was initiated by academic researchers across the North East who were keen to collaborate with health practitioners and community organisations in their research and get access to participants, data and additional funding.

These summary notes and emails were analysed in the first instance by the AskFuse Research Manager (ARM) using an auto-ethnographic approach [14]. This approach applies a cultural analysis and interpretation of the ARM’s behaviours, thoughts and experiences in relation to the policy, practice partners and researchers accessing the service. This method was chosen in recognition of the sensitive nature of the dialogues that take place between the ARM and enquirers and the importance of these dialogues for shaping collaborative research in response to their enquiries. The auto-ethnographic approach allowed for a safe deconstruction of these conversations that was sensitive to the ARM’s own input to these conversations.

The ARM read through all the notes and emails (field texts) collected from enquirers, and noted down his thoughts and reflections on barriers and facilitators for brokering enquiries through the service. The field texts facilitated the recalling and organisation of the ARM’s memories of the conversations and supported self-introspection to analyse these memories and select memories. To select memories, a series of questions was used, such as what helped to bring policy-makers and academics together and agree a protocol for a research project? What stopped some projects from advancing or academics from getting/staying involved? Memories were evaluated against these questions, searching for recurrent patterns to analyse and interpret the field texts and related memories.

The findings are based on the reflections of the ARM, which may limit generalisability to other settings. In response to this, emerging themes and categories were refined in discussions with four members of Fuse’s Translational Research programme to include a wider range of reflections and to develop his interpretations. As a group, the members reflected on the usefulness and accuracy of the themes, summarising the different experiences of the brokerage process through AskFuse and particularly the barriers and opportunities identified for collaborative working on local research projects.

While these themes adequately represent the most significant issues experienced in knowledge brokering through AskFuse, we are mindful that the service is set within an English context and therefore different themes might apply in other countries with different governance and health systems. However, the literature suggests the ubiquity of these challenges [3] and, whilst there may be much to learn from other jurisdictions where the health systems and governance arrangements may differ, some of the underlying issues that determine translation may be similar [15].

### An institutional knowledge brokering service: introducing AskFuse

AskFuse aims to respond to a broad range of research requests from the health, well-being or social care sectors. The post of AskFuse Research Manager was created to provide a single point of contact for all AskFuse enquiries and to coordinate this service for each client from start to finish. Anyone with an interest in public health (e.g. health service providers and commissioners, local government, national infrastructure organisations, and voluntary and community sector organisations) can contact the service with an enquiry, either by email, phone or by completing an online form. In an initial conversation, the ARM explores the needs of the enquirer in more detail; the nature and timescale of any further work is then agreed (with no obligation or fee), resulting in a research brief. The costs of any work are agreed, and outputs discussed at this stage. The ARM then liaises with Fuse senior investigators and staff at the five universities in the North East of England (Newcastle, Northumbria, Durham, Sunderland and Teesside) to identify capacity and skills to develop, commission, lead and undertake research projects that address the brief.

Academics are encouraged to collaborate on responsive research projects with at least two of the five universities in the Fuse collaboration when feasible. During the delivery of a project, the ARM liaises closely with the health professional(s) on the progress of the research and offers opportunities for joint reflection on the data analysis and interpretation of findings to ensure their usefulness for the enquiring organisation.

Findings from research projects and evidence synthesis brokered through AskFuse are made directly available to the health professional in short reports (20 pages) with actionable recommendations, including a one-page summary. Where appropriate and with permission from the enquirers, findings are made available to other policy-makers and health professionals through summary briefs, blogs and case studies on the Fuse website (www.fuse.ac.uk). The service is promoted through regular communication activities (e.g. press relations, newsletter, social media), presentations at events and meetings, branded materials and through a dedicated space on the Fuse website.

## Findings

### Five challenges of knowledge brokering within an institutional rapid response service

We identified five themes, representing barriers and opportunities in the knowledge brokering process through AskFuse:

1. A portal is only the start of a conversation
2. Limits to collaboration
3. It is not all about the money, but it certainly helps
4. Brokering research in a dynamic system: working with change
5. Changing evidence bases and multiple types of knowledge

#### Challenge 1: A portal is only the start of a conversation

The service aimed to create a simple, responsive ‘portal’ that would open-up the academic expertise within Fuse to policy-makers and practitioners working in public health in the North East of England. However, in many cases, enquiries appear at an early stage and it takes considerable brokerage time to develop them to the stage of being researchable projects or to determine what is required, or what would be most helpful to the enquirer. An early illustration of the working of AskFuse as a simple, linear brokerage process through one portal became, in practice, quite complex with a lengthy process of multiple, iterative conversations with various stakeholders around the enquiry and different academics within Fuse. An initial scoping of the project would raise further questions and comments from other parties, including researchers within Fuse, which would set into motion follow-up conversations to clarify the research brief. The time-consuming nature of scoping research and the short timescale often available to support decision-making have been acknowledged in other studies [2, 16].

#### Challenge 2: Limits to collaboration

As a portal, the service aimed to facilitate partnerships. However, it became clear that not all business flows through the one portal. We were aware of the presence of pre-existing partnership between individual academics and health commissioners and hoped to build on these partnerships and make them more visible across the North East for other partners. However, some partners retained their preference for working with specific individuals or institutions and did not endeavour to open-up these partnerships to other interested parties or share practices and expertise.

Moreover, there was limited capacity or willingness amongst Fuse academics – outside of a group already traditionally more service orientated – to respond to enquiries, particularly those with little monetary value. Performance-related pressures within universities means that many academics are unlikely to participate. Small-scale local projects can be difficult to translate into academic articles for peer-reviewed journals (with the exception of papers that are more practical and about collaborative experiences) and any funding secured does not carry the same prestige as a grant from a research council. This is unlikely to change unless incentive structures change within universities. Some institutions, such as Leeds University, are leading the way by including service impact or knowledge transfer as an explicit criterion for progression of their academic staff [17]. The need for better incentives to encourage universities to engage in knowledge translation has been highlighted in other studies on knowledge partnerships between researchers, practitioners, policy-makers and the commercial sector [18].

#### Challenge 3: It is not all about the money but it certainly helps

An obstacle in many conversations has been the limited availability of funding for research. It has been difficult to disabuse some enquirers (especially in voluntary and community sector organisations) who believe Fuse has a pot of money to fund research and very difficult – where people come without funds – to help them locate sufficient money to have any chance of carrying out their plans.

Consequently, the monetary value of the projects proposed is often quite small. The low value of projects, and the speed with which partners wish projects to be tendered for and then delivered, makes it difficult to accommodate many requests within the academic setting. The mismatch between policy and research timeframes and funding is also emphasised in other studies, showing that research is often published after policy decisions have been made [3, 16].

Where possible, we have supported enquirers with limited funds in identifying suitable external funding schemes and developing funding applications. However, the lengthy application and review processes of many external funders often do not match the timescales of the intervention, which in many cases has already started and, with an uncertain outcome, many enquirers are dissuaded from applying through these schemes.

More funding is now becoming available for supporting some practice-oriented work, e.g. the Public Health Practice Evaluation Scheme led by NIHR School for Public Health Research and in Public Private Partnerships with academics; however, dedicated funding schemes for applied research in public health involving co-production are still scarce.

#### Challenge 4: Brokering research in a dynamic system: working with change

A significant challenge in the development of the service was the coincidence of its launch with a major public health system upheaval brought about by the Health and Social Care Act in 2012 [19], namely the restructuring of the NHS and the move of responsibility for commissioning and delivering public health to local authorities. At that time, jobs were lost and people were moving between and out of organisations, making it difficult to maintain existing relationships or establish new ones. Given this state of flux, it has taken a long time for public health partners themselves to be comfortable with their new positions and to understand their own systems, e.g. procurement procedures within their new local authority settings.

Moreover, each organisation has developed idiosyncratically and thus there is still little consistency between local authorities in the ways in which, for instance, procurement and tendering rules are applied. Changes in the public health landscape, with the disappearance of ring-fenced budgets for public health, are likely to continue in the future and will require ongoing investment of the AskFuse service in relationship building and maintenance. The wider literature confirms that policy-makers value the credibility of researchers developed in these trusted relationships [20].

#### Challenge 5: Changing evidence bases and multiple types of knowledge

The changes to the public health system, combined with a climate of austerity and unprecedented budget cuts, confront public health professionals and academics with new and urgent questions, not only about the impact and value of their programmes but also about the evidence required to demonstrate this impact. The severity of budget cuts may mean that long-standing services need to be decommissioned to allow new developments to occur.

Public health commissioners and professionals must increasingly demonstrate the added value of their programmes and interventions to other government departments and policy areas (such as transport, education and housing). This has increased the need for investigating the evidence base around the social determinants of health. Public Health teams are also progressively tasked with designing complex interventions that draw upon a mixed evidence base and that involve working in co-production with a range of professionals outside health, such as social care workers, and volunteers working in community organisations.

At the same time, academics and researchers working in public health have explored new ways to demonstrate the impact of their research. Spurred on by the Research Excellence Framework in 2014, requiring academics, for the first time, to demonstrate the impact of their research beyond academia, researchers have developed new templates for assessing impact. This has often involved closer collaboration with research users and commissioners to define what impact is and how it could be best measured.

This provides common ground for both groups to talk about what evidence is valued and how this evidence can be best developed. For instance, some service managers might not be interested in the question ‘what works and does not work’ but would like to know how they can do the best possible job within the limited resources and time that they have. This indicates a need for a shift in evaluation away from research that simply assesses whether a programme or intervention works but rather also instigates discussion with service managers about mechanisms and ‘what works best for whom in what circumstances’. Local bodies can rarely afford expensive and lengthy trial designs that show effect. They are more likely to afford and put a higher value on the types of implementation advice that comes from qualitative or realist designs, but these have lower currency in the academic evidence hierarchy. The need to consider multiple types of evidence has been acknowledged by other studies [13], arguing that evidence considered ‘gold standard’, such as Cochrane reviews, often fail to provide direction to policy-makers on which interventions to implement and under which circumstances [21].

## Discussion: a bleak picture for institutional brokering services?

The five challenges outlined above to collaborative working on local research projects through an institutional brokerage service paint a rather bleak picture for the sustainability of these services. However, we now apply Goffman’s dramaturgical perspective to these challenges, which present them in a new light, suggesting that the challenges can be better understood as differences in performances by academics, practitioners and policy-makers that need to be effectively managed by responsive research services. In doing so, we build on what is known about these challenges in the existing literature and use a new perspective to identify how these challenges arise and could be addressed in an institutional knowledge brokering context.

### Managing performances in knowledge brokering

Services like AskFuse provide an important back stage for conversations between academics, practitioners and policy-makers, away from public view, where informal conversations can get at the heart of what policy-makers want to know or do, and what limits there might be around academics’ ability to respond to that (as outlined in Challenge 1). Time spent back stage with AskFuse helped, not only to negotiate performances, but also to decide on the staging. What is the real research question (or questions behind the question)? What type of evidence is most valued? What resources are available to conduct the research? This also helps to address Challenge 5 (Changing evidence bases) by enabling negotiation between knowledge producers and users about the type and mix of evidence that is needed for different performances.

### Performing for different audiences

As Challenge 2 highlighted, there are different audiences with whom academics and health professionals must communicate. Each group needs to present a different reality to their audience (for example, the rigour of their research, its usefulness for patients, and the cost-effectiveness of projects for commissioners) and these different realities can cause problems in the knowledge exchange process. For example, scientific rigour can clash with the timescales for developing an intervention, wherein lengthy ethics procedures that academics need to follow can draw out the research process and delay the start time of an intervention. Additionally, increasing quality of care is not always value for money. Moreover, public health interventions, no matter how effective and evidence informed, can be in direct conflict with other local interests; for instance, alcohol licensing can be perceived as a threat to the local night-time economy.

Goffman compares this to the problem of actors having to perform on different stages, giving different messages to various audiences. To keep each performance intact, ideally performers prefer to segregate their audiences so that the individuals who witness them in one role will not be the same individuals who witness them in another role, at least not simultaneously or consecutively [11]. Performing different roles is part of being a competent actor; the audience needs to believe that an actor personifies each role separately and therefore keeps the different roles separated in time across different audiences.

### When audience segregation fails

Unfortunately, Goffman points out that this is not always possible – sometimes audience segregation fails. As practitioners and academics maintain a range of networks and partnerships, their separate performances are bound to overlap and clash. Goffman proposes two solutions. First, all those in the audience may be suddenly accorded temporarily back stage status and “*collusively join the performer in abruptly shifting to an act that is fitting to the one for the intruders to observe*” [11]. In other words, the audience become performers themselves to present the right message to the new arrivals. This is done not to mislead or exclude the other audience (‘intruders’ sends the wrong message in this case), but to focus the performance on the new audience. This also avoids having to do two different performances simultaneously, which would lead to confusing messages. This solution suggests a certain amount of fluidity between audience and performers.

A second way outlined by Goffman to handle the problem of failed audience integration is to accord the intruder a clear-cut welcome as someone who should have been in the region all along and adopt them as members of the existing audience to keep the current performance on track [11].

### The back stage functions of AskFuse

The solutions outlined by Goffman for common performance problems point to various back stage functions that are provided by AskFuse. Firstly, differences in roles and audiences can be discussed back stage and more synchronised performances can be rehearsed that present the right message for the right audience at the right front stage.

Secondly, AskFuse can help with the management of ‘destructive’ information, namely messages that challenge the coherence of the different performances and discredit the impressions of the actors. To make their audiences believe in their performances, it is vital for actors to be coherent in their performance. This will require the over-communication of some facts and the under-communication of others and, for Goffman, herein lies the inherent problems that many performers face: “*There are usually facts which, if attention is drawn to them during the performance, would discredit, disrupt or make useless the impressions that the performance fosters* (destructive information)” [11]. Therefore, a basic problem for many performances is that of information control; the audience must not require ‘destructive’ information about the situation that is being defined for them. Challenge 3 highlighted that limited funding for applied research and lack of time and interest among academics can be classified as ‘destructive’ information for Fuse. It discredits or disrupts the claim that Fuse is focused on the translation of research evidence into practice and that it values collaborative working to enhance knowledge exchange. Therefore, a key function for AskFuse is how to manage this ‘destructive’ information from ruining the collaborative performances.

AskFuse provides a safe space for what Goffman calls ‘staging talk’, namely reflections between (different teams of) performers on past performances and rehearsing new ones. An important element of stage talk is ‘collective moaning’, that is moaning between different actors when back stage about past performances gone wrong, rowdy audience members and props that did not work. In order words, they share ‘destructive’ information. Goffman describes moaning as the surest sign of back stage solidarity [11]. Back stages, such as AskFuse, provide a perfect forum for a little moaning and sharing of discrediting knowledge with trusted policy and practice partners to build solidarity. In turn, this solidarity can be used for developing collaborative research projects and shared funding applications with our policy and practice partners.

In addition, AskFuse provides a medium for defusing these ‘destructive’ messages. Goffman has suggested that ‘destructive’ information can be made less harmful by ‘over-communicating’ other facts that draw attention away from these messages, for instance, by emphasising Fuse’s free Quarterly Research Meetings and Knowledge Exchange Seminars, where practitioners and policy-makers meet with academics to talk about their research and its usefulness. Alternatively, attention could be drawn to the money that has been made available through Fuse and its membership of the NIHR School of Public Health Research. The need to tailor messages to different audiences has been highlighted by other studies, suggesting that knowledge translators need to identify key messages for different target audiences [22].

### Dealing with change: ‘deviant’ roles

The back stage functions of AskFuse address some of the challenges identified in the knowledge brokering process of institutional services. However, the service does not provide a solution to rapid changes. Challenge 4 highlighted that, when health systems are restructured, the fluidity between front and back stages appears to increase, wherein new front stages are developed (for example, transfer of United Kingdom Public Health responsibility from the National Health Service to local government in 2013) and old back stage areas disappear (for example, United Kingdom Regional Health Authorities were abolished), while demarcations between audiences and performers are still unsettled. Moreover, Challenge 5 suggests that not only the stages are changing but also the types of information that need to be communicated on these stages.

To maintain (academic and health professional) performances in times of shifting contexts and stages, Goffman argues that it is necessary for actors to adopt discrepant roles and communicate out of character. One of the discrepant roles Goffman discusses is the go-between or mediator: “*The go-between learns the secrets of each side and gives each side the true impression that he will keep its secrets; but he tends to give each side the false impression that he is more loyal to it than to the other*” [11].

This role is comparable with the position of the ARM, who acts as the go-between amongst policy-makers, practitioners and academics on various shifting stages. We do not reject Goffman’s characterisation of the need to give false impressions, but we subscribe to a particular interpretation that emphasises providing different impressions to different audiences as outlined by Goffman in his description of one of the key functions of the role: “*Sometimes, the go-betweener may function as a means by which each side is given a slanted version of the other that is calculated to make a closer relationship possible*” [11].

This makes the role of the ARM not only relational but also translational – they must translate differences in performances between policy-makers, practitioners and academics into a view that is more acceptable collectively than the original projection [11]. For instance, writing a research brief together with health practitioners can help to translate initial enquiries into researchable questions that academics can relate to the existing evidence base. It helps academics to understand what evidence is valued by practitioners and helps the practitioners, in turn, to understand what research is feasible within available resources and timescales.

## Conclusions

### Key findings

Herein, we reflected on the experience of a particular knowledge-brokering model (AskFuse) developed within Fuse, the Centre for Translational Research in Public Health, in the North East of England. We identified five challenges in the brokering process of institutional services like AskFuse, related to brokerage time, scarcity of resources, lack of institutional incentives and willingness of academics to collaborate with health professionals, and ongoing structural changes in the United Kingdom health system.

Applying Goffman’s dramaturgical perspective, we reframed these challenges as differences in performances by academics, practitioners and policy-makers that need to be effectively managed by responsive research services. The AskFuse service gives these partners access to an informal conversation space that enables them to reflect on performances gone wrong, as well as to construct new impressions that will help them to cope when they act on different front stages and to different audiences.

We distinguished between different functions that responsive research services could provide in the back stage:

1. Providing a conversation space for health practitioners and academics in which to meet and engage in conversations about local research needs;
2. Discuss the different audiences with whom each actor communicates (e.g. elected members, funders, service commissioners, service users);
3. Rehearse and synchronise their performances across different stages (e.g. conferences, research events, council sessions, staff meetings);
4. Share and hide ‘destructive’ information about their performances (e.g. lack of funding, limited appetite for collaboration); and
5. Negotiate new evidence bases (e.g. affordability versus impact) by considering multiple types of evidence and applying new review [15] and research methods to make them accessible and affordable to different contexts and needs [16, 21].

In addition, knowledge broker roles in responsive research services are important to facilitate situations where audiences overlap and where back stage performers are suddenly thrown in the lime light of the front stage. Knowledge brokers can act as the go-between or mediator to translate differences in performances between policy-makers, practitioners and academics into a collective acceptable presentation.

### Limitations to a dramaturgical lens

Goffman’s dramaturgical perspective helpfully reframes the identified challenges to allow for new solutions to be explored for knowledge brokering problems identified in the literature and reiterated in this paper. However, we recognise that there are limitations to applying this metaphor to institutional knowledge brokering; not all readers will agree with some of Goffman’s solutions, such as keeping audiences separated and hiding information that could discredit performances. Knowledge brokering often aims to reach across boundaries and unite different audiences. Therefore, failed audience segregation might be more often than not a reality rather than an inconvenient anomaly for knowledge brokers. What Goffman’s dramaturgical lens helps us to do is to reflect on how this reality impacts on our performances and how we can adjust for it. Other solutions offered by Goffman appear counterintuitive at first sight, for example, temporarily hiding information that makes performances less believable. Open communication is often highlighted as an important trait of knowledge brokers; however, the dramaturgical lens suggests that sometimes being selective with the evidence and the format in which it is presented make performances more credible and knowledge brokering more effective. In strategically communicating between different parties, the knowledge broker can choose to ‘over-communicate’ some facts and ‘under-communicate’ others for different performances.

In summary, what these reflections point to is that the messiness of institutional knowledge brokering can be turned into a strength by applying a dramaturgical perspective. The messiness also means that the challenges faced by the AskFuse service will likely change over time and will require new solutions from a dramaturgical perspective, as highlighted by Challenge 4 (Working with change).

### Recommendations

As the five challenges have illustrated, institutional knowledge brokering is not a straightforward process. By understanding the dynamics of the underlying brokering process as staged performances across various stages and in front of different audiences, a more productive back stage can be created. The identified functions of responsive research services can help to develop this stage and foster collaborative partnerships between policy-makers, practitioners and academics, which ultimately increase the flow of evidence into practice.

This takes, time, resources and capacity development, which are limited in the current climate of austerity. However, the need for brokerage services is increasingly recognised by universities across the globe to facilitate the impact of their research and to encourage early conversations between academics and practitioners for developing collaborative research. Continuous investment in institutional brokerage services and research on their operation and effectiveness is required to support impact and address the challenges highlighted in this paper.
